# Research Progress on Polydopamine Nanoparticles for Tissue Engineering

**DOI:** 10.3389/fchem.2021.727123

**Published:** 2021-09-06

**Authors:** Yanmei Tang, Yu Tan, Kaili Lin, Min Zhu

**Affiliations:** ^1^ Department of Oral and Cranio-Maxillofacial Surgery, Shanghai Ninth People's Hospital, College of Stomatology, Shanghai Jiao Tong University School of Medicine; National Clinical Research Center for Oral Diseases; Shanghai Key Laboratory of Stomatology and Shanghai Research Institute of Stomatology, Shanghai, China; ^2^ Second Dental Clinic, Shanghai Ninth People's Hospital, College of Stomatology, Shanghai Jiao Tong University School of Medicine; National Clinical Research Center for Oral Diseases; Shanghai Key Laboratory of Stomatology and Shanghai Research Institute of Stomatology, Shanghai, China

**Keywords:** polydopamine, nanoparticles, biofunction, tissue engineering, tissue regeneration

## Abstract

Tissue engineering is an interdisciplinary field that aims to develop biological substitutes for the replacement, repair, or enhancement of tissue function. The physical and chemical characteristics of biomaterials exert a profound influence on the biological responses and the following biofunction. Nanostructured coatings have been widely applied as an effective surface modification strategy to improve the bioactivity of biomaterials. Especially, polydopamine and polydopamine-derived nanoparticles are found with excessive adhesiveness, redox activity, photothermal conversion capacity, paramagnetism and conductivity other than excellent biocompatibility, and hydrophilicity. In this article, advances about polydopamine nanoparticles in tissue engineering applications are reviewed, including the repair of bone, cartilage, skin, heart, and nerve, to provide strategies for future biomaterial design.

## Introduction

Tissue engineering (TE) is an interdisciplinary field that aims to develop biological substitutes for the replacement, repair or enhancement of tissue function. As one of the most important component in TE, biomaterials (or scaffolds) play the key role of stimulating and regulating targeted cells to regenerate new tissue. Generally, the physical and chemical characteristics of biomaterials exert profound influence on the biological responses and following biofunctions. Over the years, numerous strategies have been introduced to modify biomaterials for better tissue assembly and functionality. Thereinto, nanoparticles (NPs) have emerged as a powerful tool to increase the mechanical and biological properties of scaffolds. Moreover, recent researches reported extensive applications of NPs in drug/gene delivery, cell labeling/pattering, 3D tissue construction, and so on. Thus, NPs hold an importance position in biomedical field and show considerable potential in TE.

Since the debut in 2007 by Lee and Messersmith’s group (Qiu et al., 2018), polydopamine (PDA) deposition inspired by natural mussels has been developed as a facile and universal method for the surface modification of various materials. PDA can virtually deposit onto all types of materials and form functional coating surfaces. Moreover, the abundant functional moieties of PDA coatings allow for further modification to introduce bio-molecules or drugs. Thus, with the excellent adhesiveness, secondary reactivity, hydrophilicity and biocompatibility, PDA coatings are emerging and promising in TE applications.

Compared to the planar PDA coatings, the 3D PDA-NPs provide larger specific surface area to interact with more cells and bio-functional molecules. Particularly, PDA-NPs are found with unique properties such as photothermal conversion capacity and redox activities other than adhesiveness and hydrophilicity (Liu et al., 2014). As we previously reviewed, with PDA-NPs modification, excellent results have been achieved in photothermal therapy (PTT), chemical treatment, anti-inflammatory, antioxidant, and so on (Jin et al., 2020). Our previous work also proved the reactive oxygen species (ROS) scavenging facility and anti-inflammatory ability of PDA-NPs in treating temporomandibular joint osteoarthritis (TMJ-OA) (Wang et al., 2021). Moreover, various bio-functional NPs (e.g., Fe_3_O_4_, graphene oxide (GO), TiO_2_) have been reported successfully modified with PDA. Those PDA modified NPs showed excessive biocompatibility and biostability, with their original characteristics like electrical conductivity, paramagnetism, or fluorescence unaffected. Thus, PDA derived NPs have been widely investigated in biosensing, imaging, and tumor therapy (Jiang et al., 2019; Ye et al., 2020).

**FIGURE 1:**
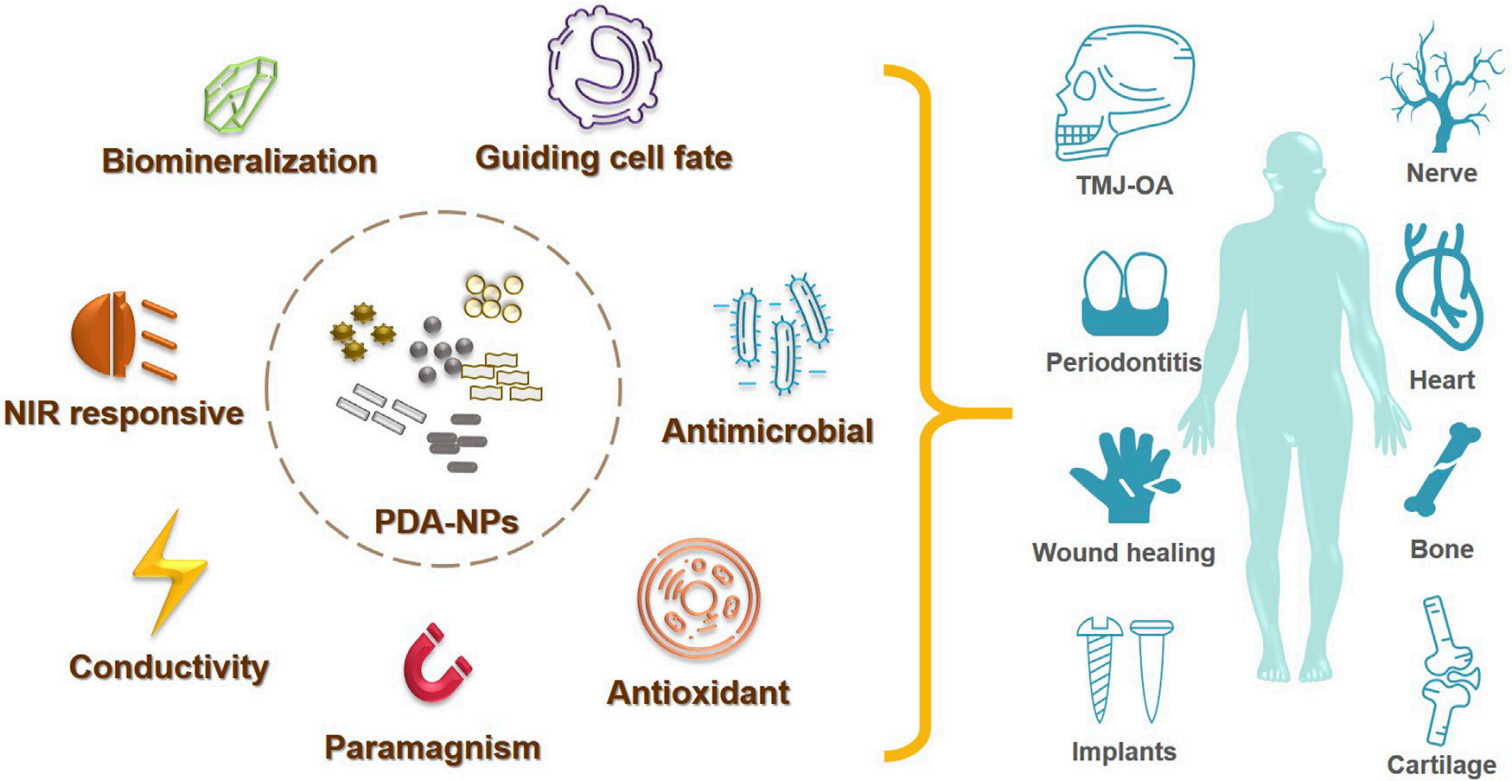
Schematic of PDA-NPs in TE application.

While significant progress has been achieved for multiple PDA-NPs applications in biomedical field, their applications in TE are still limited. This review aims at outlining the developments in the use of PDA-NPs for TE, including the fields of soft/hard tissue regeneration and biomedical implants (Figure 1). By doing so, we hope to widen the application of PDA-NPs in TE, and to enlighten our future thinking of the biomaterial designing strategies.

## Synthesis of PDA-NPs

### Process of PDA Polymerization

PDA was initially found polymerized spontaneously from dopamine-hydrochloride in the presence of alkaline buffer. Since then, the mechanism of PDA coating has been intensively studied. Until now, the mechanistic details remain elusive due to the complex involvement of numerous intermediate reactions. Nevertheless, it is now widely recognized that two steps are involved in the PDA deposition, including the oxypolymerization and the surface adhesion. The classical “eumelanin” model believes dopamine is initially oxidized to dopamine quinone (DQ). Then, the DQ undergoes cyclization and rearrangement to form intermediates such as leucodopaminechrome and dopaminechrome. Next, the key precursors, 5,6-dihydroxyindole (DHI) and indole-quinones are generated, which can further polymerize to form oligomers, finally resulting in PDA. Now, researchers propose that PDA is a complexity of covalent polymer with small molecules and oligomer components (Della Vecchia et al., 2013). Lately, with the continuous update of laboratory techniques, more models were proposed, suggesting both covalent polymerization and noncovalent self-assembly played great roles in PDA formation (Liu et al., 2014; Batul et al., 2017).

Oxygen has always been the most frequently used oxidizer to initiate the polymerization (Madhurakkat Perikamana et al., 2015). Recently, researchers reported that, other than atmospheric oxygen, metallic oxidants (copper, zinc or nickel ions) and nonmetallic oxidants (sodium periodate, ammonium persulfate, hydrogen peroxide, etc.) could both start the PDA coating (Kopec et al., 2020). Specifically, PDA can also be synthesized by electro-polymerization, exhibiting a higher deposition rate compared to the typical self-polymerization method (Wang et al., 2014). In general, the synthetic rate, coating thickness and structure, and efficiency of dopamine utilization during the PDA coating could be optimized by adjusting the process conditions or the oxidizing methods (Lee et al., 2019).

To date, the exact mechanism of the robust adhesion capability of PDA remains complicate. It’s widely accepted that multiple functional groups including catechol group, amino group, carboxylic acid group, indole units, and quinone functions contribute to the mussel-mimicking versatility of PDA. PDA coats on substrates mostly via covalent binding (Michael-type addition, Schiff base reactions) or noncovalent binding (hydrogen bonding, π−π stacking, metal chelating).

### Fabrication of PDA-NPs

PDA-NPs are developed based on the PDA coating technology to extend its utilization potential by polymerizing PDA into nanoscale particles. Essentially, PDA-NPs share the same polymerization mechanism as that of PDA coatings, except that the synthesis of PDA-NPs requires the addition of polymer inhibitors to control the polymerization speed and the particles morphology. Water-alcohol mixed solution is the most commonly used polymer inhibitor in the reaction (Yan et al., 2013). Well-dispersed PDA nanospheres would be obtained in the mixed solvents with the volume fractions of ethanol from 25 to 40% (Jiang et al., 2014). Overall, the synthesis of PDA-NPs can be controlled by tailoring the experimental parameters, such as dopamine concentration, pH, polymerization time, temperature, and so on. Here the influence factors are listed below in Table 1.

**TABLE 1:** Factors influencing the synthesis of PDA-NPs.

Factors	Influence	Ref
Monomer concentration	NPs size increases with increasing dopamine concentrations	Ju et al. (2011)
Buffer system	Particle size varies in different buffers, smaller in Tris compared to that in phosphate	Della Vecchia et al. (2014)
Temperature	The reaction rate increases by increasing the temperature, leading to smaller particle size with higher density	(Ju et al., 2011; Cho and Kim, 2015)
Reaction time	Increasing reaction time would enhance the deposition of dopamine	Jiang et al. (2011)
Oxidants	Oxidants like sodium periodate or potassium chlorate would speed up the polymerization in alkaline aqueous media	Wei et al. (2010)
pH	Higher pH conditions result in a higher yield of particles with smaller diameters	Ho and Ding, (2013)
Metal ion additives	Metal ions facilitate dopamine polymerization	Wang et al. (2017b)
Free radicals	Free radical scavengers inhibit NPs growth while stable free radicals facilitate seed formation. Both decrease NPs size	Wang et al. (2019b)
Diads in proteins	Diads increase PDA formation to obtain biocompatible PDA@protein NPs	El Yakhlifi and Ball, (2020)

To increase the consistency and homogeneity of PDA polymerization, several methods have been mentioned. Kim et al. claimed high O_2_ concentrations in the dopamine solution lead to highly homogeneous layer deposition on substrate surfaces with accelerated reaction rate (Kim et al., 2013). Ponzio et al. found periodate oxidant promoted fast and homogeneous deposition of PDA with thickness up to 100 nm (Ponzio et al., 2016). Fan et al. reported folic acid influenced PDA nanostructures via enhancing the π−π interactions of oligomers (Fan et al., 2015).

Owing to the abundant functional groups (catechol and phenethylamine), PDA exhibits high positive bioactivity. To date, PDA has been successfully coated/modified on various kinds of nanostructures, including the organic and inorganic NPs, nanotubes (NTs) and nanorods, such as liposomes (Awasthi et al., 2020), polymeric NPs (Zhu et al., 2016), magnetic NPs (Oroujeni et al., 2018), silica NPs (Liu et al., 2018), gold NPs (Sy et al., 2018), carbon NTs (Qu et al., 2017), and so on. Meanwhile, PDA nanospheres also serve as active and facile templates for synthesis of hollow or core/shell nanostructures (Yan et al., 2013; Tran et al., 2019). This part was previously reviewed by our team in detail (Jin et al., 2020). By template/solvent method, PDA nano/microcapsules were obtained, exhibiting outstanding unidirectional loading and release behavior (Yu et al., 2009). Thus, PDA-NPs provide a versatile platform for multifunctional biomedical applications. With active hydroxy and amino surface groups, PDA-NPs gain excessive biofunctions by immobilizing cells, DNA, proteins, drugs, and minerals.

## Biomedical Applications of PDA-NPs in TE

TE requires a series of design and optimization of biomaterials for better repair, restore or regenerate the damaged tissue. As the preliminary step in TE, cell adhesion plays a crucial role in regulating cellular functions. Various researches have reported the PDA (or PDA-NPs) coatings on scaffolds/implants would promote cell spreading, proliferation and migration owing to their outstanding hydrophilicity and biocompatibility (Li et al., 2017; Jin et al., 2020). Recently, more studies focus on exploring other characteristics of PDA-NPs and PDA-derived NPs in TE, such as differentiation promoting, antibacterial and antioxidant specialty, as well as photothermal property. Here we exemplified those applications in Table 2 and reviewed in the following.

**TABLE 2:** Examples for PDA-NPs application in TE.

Object	Form	Average size	Cell culture	Property	TE application	Ref
PDA-MSNPs	Oval shape	120 nm	Pancreatic islets	Biocompatibility	Renal subcapsule islet transplantation in diabetic mice	Razavi et al. (2020)
PDA/PCL fibers	Nanofibers	180–220 nm	Human BMSCs	Cell differentiation and biomineralization promotion	Bone defect on mouse skull	Deng et al. (2019)
PDA/HA NPs	Nanorods	186 ± 6 nm	BMSCs	Surface coating for cytokine adhesion	Ti implants for rat femoral bone regeneration	Wang et al. (2017a)
PDA@ZnO NPs	Nanorods	218 nm (coating of 23 nm)	L929 fibroblasts	Antibacterial, hemostatic potential	*In vitro* (for wound healing)	Tavakoli et al. (2021)
PDA-NPs	Spherical structure	117.7 nm	Rat chondrocytes	ROS scavengers	Rat TMJ-OA	Wang et al. (2021)
PDA-NPs	Spherical structure	160 nm	HGE cells	ROS scavengers	LPS-induced periodontitis in mice	Bao et al. (2018)
CQD/ZnO-PDA NPs	Particles	100 nm	NIH3T3 cells	NIR responsiveness	Rat dorsal wound model	Xiang et al. (2019)
PDA-rGO NPs	Nanosheets	50–200 nm	Rat cardiomyocytes	Electric conductivity	*In vitro* constructing cardiac microtissue	Li et al. (2021)
Fe_3_O_4_@PDA NPs	Spherical structure	55–60 nm	Human umbilical cord MSCs	Paramagnetic responsiveness	Sciatic nerve chronic compression injury model in rats	Liu et al. (2021)

### Guiding Cell Behavior

Degradable scaffolds and membranes derived from various polymers show grate advantages in guided TE. Whereas, limited by the hydrophobic and bioinert nature, synthesized polymers exhibit poor cell attachment or tissue integration (Talon et al., 2019).

In the work of Deng et al. (Deng et al., 2019), PDA-NPs were optimized to coat on the electrospun membrane of polycaprolactone (PCL) fibers. It turned out that, not only the cell attachment and proliferation, but also the osteo-differentiation of human mesenchymal stem cells (hMSCs) were prominently improved. Additionally, PDA-NPs are endowed with superior affinity to various kinds of proteins. Wang et al. (Wang et al., 2016) proved bone morphogenetic protein-2 (BMP-2) was absorbed on PDA-NPs modified scaffolds and sustainably released *in vitro* up to 30d. Thus, PDA-NPs immobilization accelerated new bone formation in porous scaffold subcutaneously implanted *in vivo*. Moreover, a silica NP coated with PDA (PDA/SiNP) was developed for hemostasis (Liu et al., 2018). PDA/SiNP showed appropriate hydrophobicity, and promoted erythrocytes aggregation. It remarkably accelerated coagulation, proved to be an excellent hemostat in future hemorrhage treatment.

To conclude, by mimicking the nano-topographical and bio-chemical clues of extracellular matrix (ECM), biomaterials with specific properties are capable of guiding cell fate. PDA-NPs provide promising directions for future materials design.

### Antimicrobial Activity

PDA-NPs are now emerging in the field of antibacterial application, particularly with the wound healing in TE. Fu et al. (Fu et al., 2021)found that treating PDA-NPs with ascorbic acid was a simple strategy to obtain reduced PDA-NPs (rPDA-NPs), which showed improved antioxidative and antibacterial activity. Thus, the oxidized dextran/chitosan composite hydrogel incorporated with rPDA-NPs promoted the healing of infected full-thickness wound in rat dorsal skin.

**FIGURE 2:**
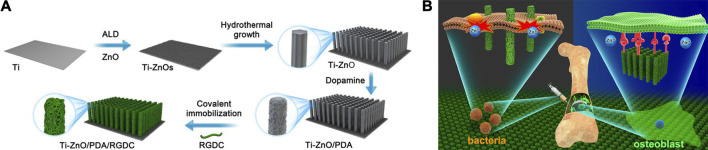
Ti implants modified with ZnO/PDA/RGDC nanorods balance the bacteria−osteoblast competition through selective physical puncture (Li et al., 2017).

Simultaneously, researches about NPs modified by PDA as novel antimicrobial agents have also sprung up. To date, PDA has been reported successfully coated on various NPs, including Cu/Ag hybrid metal core (Yeroslavsky et al., 2016), TiO_2_ nanotubes (Xu et al., 2017), ZnO nanorods (Tavakoli et al., 2021), GO (Liang et al., 2019a), carbon nanotubes (CNT) (Liang et al., 2019b) and so on. Li et al. (Li et al., 2017) introduced enhanced antibacterial ability to Ti implant via designing hybrid ZnO/PDA/arginine-glycine-aspartic acid-cysteine (RGDC) nanorod arrays. ZnO/PDA/RGDC nanorods could effectively kill bacteria through physical puncture without damaging the osteoblasts (Figure 2).

PDA coating effectively increased the hydrophilicity and dispersity of NPs while reduced their cytotoxicity. Both *in vitro* and *in vivo* experiments demonstrated composite biomaterials engineered by PDA modified NPs exhibited gratifying antibacterial properties. Possible mechanisms can be attributed to reactive oxygen species (ROS) or adenosine triphosphate (ATP) depletion, biomolecule interaction and regulation, and membrane interaction (Slavin et al., 2017; Jiang et al., 2020). As a result, in the background of antibiotics resistance, PDA related NPs are promising alternatives for clinical control and treatment of infection, in addition to the antimicrobial biomaterials and dressings construction (Alves et al., 2019).

In addition, PDA NPs possess excellent capacity for loading drugs i.e., antibiotic. Batul et al. (Batul et al., 2020) investigated an *in-situ* polymerization method to load gentamicin into PDA NPs. The antibiotic loaded PDA-NPs are very promising for the long term drug release microbial infection treating.

### Antioxidant and Anti-inflammatory Activity

The overproduction of ROS is related to cellular dysfunction and hindered tissue recovery. To protect neurons from the oxidative stress-induced damage, Battaglini et al. (Battaglini et al., 2020) proposed to use lipid coated PDA-NPs (L-PDNPs) as neuroprotective agents. L-PDNPs were proved effective in reducing ROS accumulation and resisting mitochondrial morphological alteration.

**FIGURE 3:**
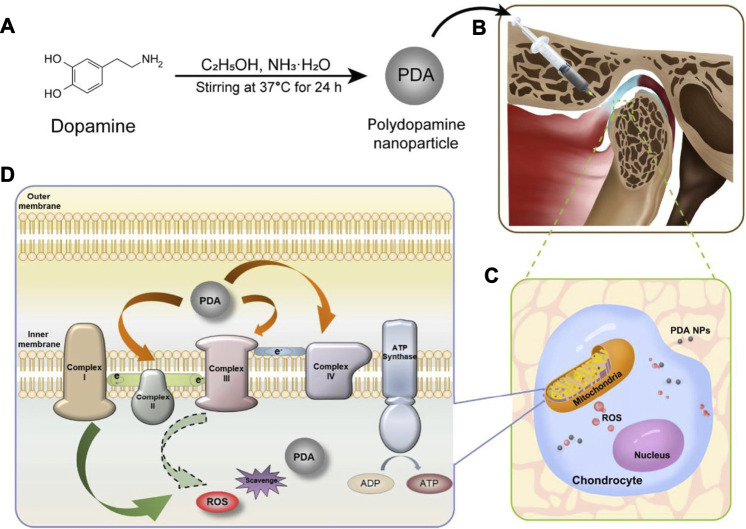
PDA-NPs showed antioxidative and anti-inflammatory dual ability in the rat TMJ-OA model (Wang et al., 2021).

In our recent work (Wang et al., 2021), PDA-NPs were found act as ROS scavenger by both directly reacting with ROS and indirectly reducing ROS production via increasing the efficiency of mitochondrial oxidative phosphorylation (Figure 3). Meanwhile, PDA-NPs downregulated the repression of proinflammatory cytokines in chondrocytes and relived the inflammation of cartilage and subchondral bone in the rat TMJ-OA model. The charming antioxidative and anti-inflammatory dual ability of PDA-NPs holds great potential for future ROS-responsive biomaterial and biosystem design (Sui et al., 2020).

### Promoting Biomineralization and Osseointegration

Biomineralization generates lays of calcium phosphate (CaP) crystals on substrates, and induces osteogenesis during bone regeneration. PDA has been proved efficient in facilitating hydroxyapatite (HA) formation on scaffolds owing to the plentiful catecholamine groups of its structure (Ghorbani et al., 2019b). Wang et al. (Wang et al., 2017a) used PDA-NPs and HA nanorods to prepare hierarchical micro-/nano-structured coating on Ti implants. *In vivo* evaluation showed improved osseointegration around Ti bars implanted in the bone marrow cavity of rats.

Previously, our work (Wang et al., 2019) with PDA coating on different implants also proved enhance osteointegration and accelerated new bone formation via PDA coating. Moreover, we found that focal adhesion kinase (FAK) and p38 signaling pathways played important roles in the osteogenic differentiation of BMSCs stimulated by PDA coatings.

Ghorbani et al. (Ghorbani et al., 2019a) found that using microwave irradiation would accelerated HA mineralization on PDA spheres. Meanwhile, the mineralized PDA spheres promoted cell adhesion and spreading better than pure PDA.

### Near-Infrared (NIR) Irradiation Responsiveness and Thermal Therapy

**FIGURE 4:**
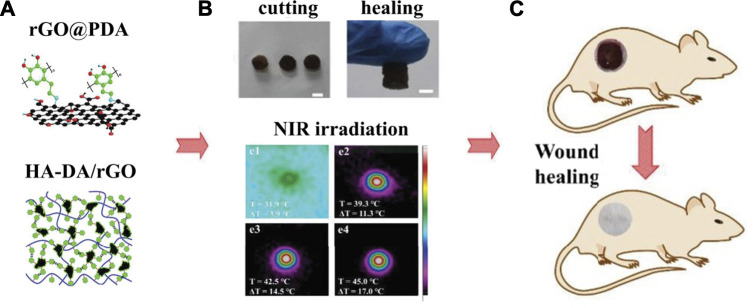
Adhesive injectable composite HA-DA/rGO hydrogels with photothermal antibacterial activity promoted skin regeneration in mouse wound healing (Liang et al., 2019a).

PDA-NPs possess excellent photothermal conversion property that could efficiently convert NIR light into heat. Both Han and Tao et al. (Han et al., 2016; Tao et al., 2021) used PDA-NPs to prepare NIR light stimuli-responsive hydrogels. Enhanced elasticity, biocompatibility, tissue-adhesiveness, and more importantly, bacteria-killing ability by photothermal effect were achieved in the composite hydrogels, showing promising future as wound dressings to accelerate skin tissue repair. Moreover, Liang et al. (Liang et al., 2019a) used hyaluronic acid (HA)-graft-dopamine and PDA decorated-reduced GO (rGO) to prepare adhesive and photothermal composite hydrogels. NIR irradiation enhanced the antibacterial performance of these hydrogels, achieving better healing outcomes compared to the commercial films in the mouse wound model (Figure 4).

Interestingly, Huang et al. (Huang et al., 2017) found PDA-NPs worked as artificial microparasols to mimic melanosomes for protecting epidermal keratinocytes from UV damage. Thus, PDA-NPs showed potential in developing novel natural melanin replacement therapies in diseases (e.g., skin cancer, vitiligo, and albinism).

Recently, plenty researched have reported new designs of biomaterials for tumor thermal ablation therapy using the feasible PDA/PDA-NPs platform. Tan et al. (Tan et al., 2016) constructed a novel PDA-NP loading ionic liquids to work as microwave susceptible agent. The NPs showed excellent microwave heating efficiency and were quite potential for tumor microwave thermal therapy. More investigations are expected in the future biomaterial design considering the photothermal or microwave thermal conversion capacity of PDA-NPs.

### Electric Conductivity for Enhanced Tissue Regeneration

PDA displays water-dependent semiconductor-like optoelectronic property. More recently, its conductivity is explained by a chemical disorder model, including a dynamic component of reversible intermolecular interactions perturbing π-electron systems (Liu et al., 2014; Pezzella et al., 2015). More importantly, PDA provides a versatile platform for suitable substrates to tuning their electronic properties.

The conductivity of polymers shows beneficial effects on the repair and regeneration of damaged tissues (Chen et al., 2021). Li et al. (Li et al., 2021) used PDA to prepare reduced GO (rGO), making graphene nano-sheets to be conductive and disperse more easily. Gelatin methacrylate (GelMA) hydrogels incorporated with PDA-rGO nanocomposites facilitated the fast maturation of cardiomyocytes, acting as an ideal candidate for cardiac TE application.

### Paramagnetic Responsiveness to Promote Tissue Repair

Recent strategies in TE now gradually recognize the magnetic responsiveness of scaffolds in an external magnetic field. Co-deposit of Fe_3_O_4_ NPs and PDA on the surface of titanium scaffolds was found to enhance the osteogenic differentiation of MSCs and new bone formation *in vivo* with a static magnetic field (Huang et al., 2020).

Furthermore, magnetic NPs are employed in disease diagnose and treatment (Jia et al., 2020). Ouyang et al. (Ouyang et al., 2019) prepared a novel magnetic resonance contrast NPs with PDA coating which were cartilage-specific and magnetic resonance imaging (MRI) contrast. The composite NPs could protect chondrocytes from apoptosis and inflammation via TLR-2/NF-κB/Akt signaling, and are quite potential for future OA treatment.

## Conclusion and Outlook

Inspired by mussel adhesion, PDA and PDA-NPs coatings exhibit excellent biocompatibility, hydrophilicity and adhesiveness. With abundant moieties, PDA-NPs can virtually functionalize any inert biomaterial surfaces to provide specific platforms for stem cells. By adjusting synthesis parameters or the oxidizing methods, the diameter and morphology of PDA-NPs can be optimized to achieve better physical and chemical properties.

During the last decade, researchers focused more on the characteristics of antimicrobial effect, antioxidant/anti-inflammation activity, conductivity, NIR and magnetic responsiveness of PDA-NPs and PDA derived NPs. A broad spectrum of TE applications using PDA related NPs has been reported in both soft and hard tissue defects model, including the bone, cartilage, skin, heart, nerve, dental tissue and so on.

As our knowledge about the features of NPs and PDA increases, new intelligent biomaterials with sequential biofunctions can be synthesized to meet specific needs during biomedical applications, for example, for the wound healing of diabetic patients or the elderly. In addition, the range of TE application could also be widened considering the fluorescent property of PDA-NPs, as well as their potentials in the construction of 3D engineered tissues via magnetic cell patterning/seeding.

However, future challenges still stand in front. As we know, PDA-NPs have been proved biocompatible with multiple human and mouse cells. Neither any dysfunction nor morphological change was found in kidney, spleen, lung, liver or heart 1 month after PDA-NPs injection into rats (Hauser et al., 2020). PDA-NPs are believed biodegradable by H_2_O_2_ and free radical generated by animal body. Still, more detailed investigation and complete evaluation are required to reach the thorough understanding of PDA-NPs degradation. So far, well-designed preclinical and clinical trials about PDA-NPs in TE haven’t been reported. The biosafety and long-term toxicity should be examined in human bodies. Another further breakthrough lies in the accurate interpretation of the PDA polymerization process. By figuring out the structural determinants of the dark brown coating, we would be able to adjust the color of PDA-NPs coatings for better wearing experience, especially in wound dressings. In this way, it is expected to propel the development of PDA-NPs derived hybrid materials forward from labs to practical and clinical use.

## Abbreviations

BMSC, bone marrow mesenchymal stem cell; CDQ, carbon quantum dots; HA, hydroxyapatite; HGE, human gingival epithelial; LPS, lipopolysaccharide; MSNP, mesoporous silica nanoparticle; NIR, near-infrared; PCL, polycaprolactone; rGO, reduced graphene oxide; ROS, reactive oxygen species; TE, tissue engineering; TMJ-OA, temporomandibular joint osteoarthritis.

## References

[B1] Alves D. Vaz A. T. Grainha T. Rodrigues C. F. Pereira M. O. (2019). Design of an Antifungal Surface Embedding Liposomal Amphotericin B through a Mussel Adhesive-Inspired Coating Strategy. Front. Chem. 7, 431. 10.3389/fchem.2019.00431 31275922 PMC6591271

[B2] Awasthi A. K. Gupta S. Thakur J. Gupta S. Pal S. Bajaj A. (2020). Polydopamine-on-liposomes: Stable Nanoformulations, Uniform Coatings and superior Antifouling Performance. Nanoscale 12, 5021–5030. 10.1039/c9nr07770g 32065189

[B3] Bao X. Zhao J. Sun J. Hu M. Yang X. (2018). Polydopamine Nanoparticles as Efficient Scavengers for Reactive Oxygen Species in Periodontal Disease. ACS Nano 12, 8882–8892. 10.1021/acsnano.8b04022 30028940

[B4] Battaglini M. Marino A. Carmignani A. Tapeinos C. Cauda V. Ancona A. (2020). Polydopamine Nanoparticles as an Organic and Biodegradable Multitasking Tool for Neuroprotection and Remote Neuronal Stimulation. ACS Appl. Mater. Inter. 12, 35782–35798. 10.1021/acsami.0c05497 PMC8009471 32693584

[B5] Batul R. Bhave M. P. J. M. Yu A. (2020). Polydopamine Nanosphere with *In-Situ* Loaded Gentamicin and its Antimicrobial Activity. Molecules 25, 2090. 10.3390/molecules25092090 PMC7250025 32365745

[B6] Batul R. Tamanna T. Khaliq A. Yu A. (2017). Recent Progress in the Biomedical Applications of Polydopamine Nanostructures. Biomater. Sci. 5, 1204–1229. 10.1039/c7bm00187h 28594019

[B7] Chen Q. Feng L. Cheng H. Wang Y. Wu H. Xu T. (2021). Mussel-inspired Ultra-stretchable, Universally Sticky, and Highly Conductive Nanocomposite Hydrogels. J. Mater. Chem. B 9, 2221–2232. 10.1039/d1tb00019e 33623949

[B8] Cho S. Kim S.-H. (2015). Hydroxide Ion-Mediated Synthesis of Monodisperse Dopamine-Melanin Nanospheres. J. Colloid Interf. Sci. 458, 87–93. 10.1016/j.jcis.2015.06.051 26210098

[B9] Della Vecchia N. F. Avolio R. Alfè M. Errico M. E. Napolitano A. D'ischia M. (2013). Building-Block Diversity in Polydopamine Underpins a Multifunctional Eumelanin-type Platform Tunable through a Quinone Control Point. Adv. Funct. Mater. 23, 1331–1340. 10.1002/adfm.201202127

[B10] Della Vecchia N. F. Luchini A. Napolitano A. D’Errico G. Vitiello G. Szekely N. (2014). Tris Buffer Modulates Polydopamine Growth, Aggregation, and Paramagnetic Properties. Langmuir 30, 9811–9818. 10.1021/la501560z 25066905

[B11] Deng Y. Yang W.-Z. Shi D. Wu M. Xiong X.-L. Chen Z.-G. (2019). Bioinspired and Osteopromotive Polydopamine Nanoparticle-Incorporated Fibrous Membranes for Robust Bone Regeneration. NPG Asia Mater. 11. 10.1038/s41427-019-0139-5

[B12] El Yakhlifi S. Ball V. (2020). Polydopamine as a Stable and Functional Nanomaterial. Colloids Surf. B: Biointerfaces 186, 110719. 10.1016/j.colsurfb.2019.110719 31846893

[B13] Fan H. Yu X. Liu Y. Shi Z. Liu H. Nie Z. (2015). Folic Acid-Polydopamine Nanofibers Show Enhanced Ordered-Stacking via π-π Interactions. Soft Matter. 11, 4621–4629. 10.1039/c5sm00732a 25959650

[B14] Fu Y. Zhang J. Wang Y. Li J. Bao J. Xu X. (2021). Reduced Polydopamine Nanoparticles Incorporated Oxidized Dextran/chitosan Hybrid Hydrogels with Enhanced Antioxidative and Antibacterial Properties for Accelerated Wound Healing. Carbohydr. Polym. 257, 117598. 10.1016/j.carbpol.2020.117598 33541635

[B15] Ghorbani F. Zamanian A. Behnamghader A. Daliri-Joupari M. (2019a). Bone-like Hydroxyapatite Mineralization on the Bio-Inspired PDA Nanoparticles Using Microwave Irradiation. Surf. Inter. 15, 38–42. 10.1016/j.surfin.2019.01.007

[B16] Ghorbani F. Zamanian A. Behnamghader A. Joupari M. D. (2019b). A Facile Method to Synthesize Mussel-Inspired Polydopamine Nanospheres as an Active Template for *In Situ* Formation of Biomimetic Hydroxyapatite. Mater. Sci. Eng. C 94, 729–739. 10.1016/j.msec.2018.10.010 30423759

[B17] Han L. Zhang Y. Lu X. Wang K. Wang Z. Zhang H. (2016). Polydopamine Nanoparticles Modulating Stimuli-Responsive PNIPAM Hydrogels with Cell/Tissue Adhesiveness. ACS Appl. Mater. Inter. 8, 29088–29100. 10.1021/acsami.6b11043 27709887

[B18] Hauser D. Septiadi D. Turner J. Petri-Fink A. Rothen-Rutishauser B. (2020). From Bioinspired Glue to Medicine: Polydopamine as a Biomedical Material. Materials (Basel) 13, 1730. 10.3390/ma13071730 PMC7178714 32272786

[B19] Ho C.-C. Ding S.-J. (2013). The pH-Controlled Nanoparticles Size of Polydopamine for Anti-cancer Drug Delivery. J. Mater. Sci. Mater. Med. 24, 2381–2390. 10.1007/s10856-013-4994-2 23797829

[B20] Huang Y. Li Y. Hu Z. Yue X. Proetto M. T. Jones Y. (2017). Mimicking Melanosomes: Polydopamine Nanoparticles as Artificial Microparasols. ACS Cent. Sci. 3, 564–569. 10.1021/acscentsci.6b00230 28691067 PMC5492417

[B21] Huang Z. He Y. Chang X. Liu J. Yu L. Wu Y. (2020). A Magnetic Iron Oxide/Polydopamine Coating Can Improve Osteogenesis of 3D-Printed Porous Titanium Scaffolds with a Static Magnetic Field by Upregulating the TGFβ-Smads Pathway. Adv. Healthc. Mater. 9, e2000318. 10.1002/adhm.202000318 32548975

[B22] Jia C. Wu H. Luo K. Hao W. Wang S. Huang M. (2020). Magnetic Silica Nanosystems with NIR-Responsive and Redox Reaction Capacity for Drug Delivery and Tumor Therapy. Front. Chem. 8, 567652. 10.3389/fchem.2020.567652 33195055 PMC7643033

[B23] Jiang J. Zhu L. Zhu L. Zhu B. Xu Y. (2011). Surface Characteristics of a Self-Polymerized Dopamine Coating Deposited on Hydrophobic Polymer Films. Langmuir 27, 14180–14187. 10.1021/la202877k 22011109

[B24] Jiang S. Lin K. Cai M. (2020). ZnO Nanomaterials: Current Advancements in Antibacterial Mechanisms and Applications. Front. Chem. 8, 580. 10.3389/fchem.2020.00580 32793554 PMC7385224

[B25] Jiang X. Wang Y. Li M. (2014). Selecting Water-Alcohol Mixed Solvent for Synthesis of Polydopamine Nano-Spheres Using Solubility Parameter. Sci. Rep. 4, 6070. 10.1038/srep06070 25317902 PMC4677634

[B26] Jiang Y. Tang Y. Miao P. (2019). Polydopamine Nanosphere@silver Nanoclusters for Fluorescence Detection of Multiplex Tumor Markers. Nanoscale 11, 8119–8123. 10.1039/c9nr01307e 30994693

[B27] Jin A. Wang Y. Lin K. Jiang L. (2020). Nanoparticles Modified by Polydopamine: Working as "drug" Carriers. Bioactive Mater. 5, 522–541. 10.1016/j.bioactmat.2020.04.003 PMC7170807 32322763

[B28] Ju K.-Y. Lee Y. Lee S. Park S. B. Lee J.-K. (2011). Bioinspired Polymerization of Dopamine to Generate Melanin-like Nanoparticles Having an Excellent Free-Radical-Scavenging Property. Biomacromolecules 12, 625–632. 10.1021/bm101281b 21319809

[B29] Kim H. W. Mccloskey B. D. Choi T. H. Lee C. Kim M.-J. Freeman B. D. (2013). Oxygen Concentration Control of Dopamine-Induced High Uniformity Surface Coating Chemistry. ACS Appl. Mater. Inter. 5, 233–238. 10.1021/am302439g 23273315

[B30] Kopec K. Wojasinski M. Ciach T. (2020). Superhydrophilic Polyurethane/Polydopamine Nanofibrous Materials Enhancing Cell Adhesion for Application in Tissue Engineering. Int. J. Mol. Sci. 21 (8), 6798. 10.3390/ijms21186798 PMC7555238 32947971

[B31] Lee H. A. Ma Y. Zhou F. Hong S. Lee H. (2019). Material-Independent Surface Chemistry beyond Polydopamine Coating. Acc. Chem. Res. 52, 704–713. 10.1021/acs.accounts.8b00583 30835432

[B32] Li B.-C. Ren K.-F. Zhang H. Jia F. Wang J.-L. Chang H. (2017a). Nanostructured Multilayer Films Assembled from Poly(dopamine)-Coated Carbon Nanotubes for Controlling Cell Behavior. ChemNanoMat. 3, 319–327. 10.1002/cnma.201700024

[B33] Li J. Tan L. Liu X. Cui Z. Yang X. Yeung K. W. K. (2017b). Balancing Bacteria-Osteoblast Competition through Selective Physical Puncture and Biofunctionalization of ZnO/Polydopamine/Arginine-Glycine-Aspartic Acid-Cysteine Nanorods. ACS Nano 11, 11250–11263. 10.1021/acsnano.7b05620 29049874

[B34] Li X.-P. Qu K.-Y. Zhou B. Zhang F. Wang Y.-Y. Abodunrin O. D. (2021). Electrical Stimulation of Neonatal Rat Cardiomyocytes Using Conductive Polydopamine-Reduced Graphene Oxide-Hybrid Hydrogels for Constructing Cardiac Microtissues. Colloids Surf. B: Biointerfaces 205, 111844. 10.1016/j.colsurfb.2021.111844 34015732

[B35] Liang Y. Zhao X. Hu T. Chen B. Yin Z. Ma P. X. (2019a). Adhesive Hemostatic Conducting Injectable Composite Hydrogels with Sustained Drug Release and Photothermal Antibacterial Activity to Promote Full-Thickness Skin Regeneration during Wound Healing. Small 15, e1900046. 10.1002/smll.201900046 30786150

[B36] Liang Y. Zhao X. Hu T. Han Y. Guo B. (2019b). Mussel-inspired, Antibacterial, Conductive, Antioxidant, Injectable Composite Hydrogel Wound Dressing to Promote the Regeneration of Infected Skin. J. Colloid Interf. Sci. 556, 514–528. 10.1016/j.jcis.2019.08.083 31473541

[B37] Liu C. Yao W. Tian M. Wei J. Song Q. Qiao W. (2018). Mussel-inspired Degradable Antibacterial Polydopamine/silica Nanoparticle for Rapid Hemostasis. Biomaterials 179, 83–95. 10.1016/j.biomaterials.2018.06.037 29980077

[B38] Liu M. Yu W. Zhang F. Liu T. Li K. Lin M. (2021). Fe3O4@Polydopamine-Labeled MSCs Targeting the Spinal Cord to Treat Neuropathic Pain under the Guidance of a Magnetic Field. J. Nanomedicine. 16, 3275–3292. 10.2147/ijn.s296398 PMC8123975 34007177

[B39] Liu Y. Ai K. Lu L. (2014). Polydopamine and its Derivative Materials: Synthesis and Promising Applications in Energy, Environmental, and Biomedical fields. Chem. Rev. 114, 5057–5115. 10.1021/cr400407a 24517847

[B40] Madhurakkat Perikamana S. K. Lee J. Lee Y. B. Shin Y. M. Lee E. J. Mikos A. G. (2015). Materials from Mussel-Inspired Chemistry for Cell and Tissue Engineering Applications. Biomacromolecules 16, 2541–2555. 10.1021/acs.biomac.5b00852 26280621

[B41] Oroujeni M. Kaboudin B. Xia W. Jönsson P. Ossipov D. A. (2018). Conjugation of Cyclodextrin to Magnetic Fe3O4 Nanoparticles via Polydopamine Coating for Drug Delivery. Prog. Org. Coat. 114, 154–161. 10.1016/j.porgcoat.2017.10.007

[B42] Ouyang Z. Tan T. Liu C. Duan J. Wang W. Guo X. (2019). Targeted Delivery of Hesperetin to Cartilage Attenuates Osteoarthritis by Bimodal Imaging with Gd2(CO3)3@PDA Nanoparticles via TLR-2/nf-κB/Akt Signaling. Biomaterials 205, 50–63. 10.1016/j.biomaterials.2019.03.018 30903825

[B43] Pezzella A. Barra M. Musto A. Navarra A. Alfè M. Manini P. (2015). Stem Cell-Compatible Eumelanin Biointerface Fabricated by Chemically Controlled Solid State Polymerization. Mater. Horiz. 2, 212–220. 10.1039/c4mh00097h

[B44] Ponzio F. Barthès J. Bour J. Michel M. Bertani P. Hemmerlé J. (2016). Oxidant Control of Polydopamine Surface Chemistry in Acids: A Mechanism-Based Entry to Superhydrophilic-Superoleophobic Coatings. Chem. Mater. 28, 4697–4705. 10.1021/acs.chemmater.6b01587

[B45] Qiu W.-Z. Yang H.-C. Xu Z.-K. (2018). Dopamine-assisted Co-deposition: An Emerging and Promising Strategy for Surface Modification. Adv. Colloid Interf. Sci. 256, 111–125. 10.1016/j.cis.2018.04.011 29776584

[B46] Qu K. Zheng Y. Jiao Y. Zhang X. Dai S. Qiao S. Z. (2017). Polydopamine‐Inspired, Dual Heteroatom‐Doped Carbon Nanotubes for Highly Efficient Overall Water Splitting. Adv. Energ. Mater. 7, 1602068. 10.1002/aenm.201602068

[B47] Razavi M. Primavera R. Kevadiya B. D. Wang J. Ullah M. Buchwald P. (2020). Controlled Nutrient Delivery to Pancreatic Islets Using Polydopamine-Coated Mesoporous Silica Nanoparticles. Nano Lett. 20, 7220–7229. 10.1021/acs.nanolett.0c02576 32909757 PMC8121116

[B48] Slavin Y. N. Asnis J. Häfeli U. O. Bach H. (2017). Metal Nanoparticles: Understanding the Mechanisms behind Antibacterial Activity. J. Nanobiotechnol 15, 65. 10.1186/s12951-017-0308-z PMC5627441 28974225

[B49] Sui L. Wang J. Xiao Z. Yang Y. Yang Z. Ai K. (2020). ROS-scavenging Nanomaterials to Treat Periodontitis. Front. Chem. 8, 595530. 10.3389/fchem.2020.595530 33330384 PMC7672210

[B50] Sy K. H. S. Ho L. W. C. Lau W. C. Y. Ko H. Choi C. H. J. (2018). Morphological Diversity, Protein Adsorption, and Cellular Uptake of Polydopamine-Coated Gold Nanoparticles. Langmuir 34, 14033–14045. 10.1021/acs.langmuir.8b02572 30360612

[B51] Talon I. Schneider A. Ball V. Hemmerle J. (2019). Polydopamine Functionalization: A Smart and Efficient Way to Improve Host Responses to E-PTFE Implants. Front. Chem. 7, 482. 10.3389/fchem.2019.00482 31338362 PMC6629787

[B52] Tan L. Tang W. Liu T. Ren X. Fu C. Liu B. (2016). Biocompatible Hollow Polydopamine Nanoparticles Loaded Ionic Liquid Enhanced Tumor Microwave Thermal Ablation *In Vivo* . ACS Appl. Mater. Inter. 8, 11237–11245. 10.1021/acsami.5b12329 27089478

[B53] Tao B. Lin C. Yuan Z. He Y. Chen M. Li K. (2021). Near Infrared Light-Triggered On-Demand Cur Release from Gel-PDA@Cur Composite Hydrogel for Antibacterial Wound Healing. Chem. Eng. J. 403, 126182. 10.1016/j.cej.2020.126182

[B54] Tavakoli S. Kharaziha M. Nemati S. (2021). Polydopamine Coated ZnO Rod-Shaped Nanoparticles with Noticeable Biocompatibility, Hemostatic and Antibacterial Activity. Nano-structures. Nano-objects 25, 100639. 10.1016/j.nanoso.2020.100639

[B55] Tran H. Q. Bhave M. Xu G. Sun C. Yu A. (2019). Synthesis of Polydopamine Hollow Capsules via a Polydopamine Mediated Silica Water Dissolution Process and its Application for Enzyme Encapsulation. Front. Chem. 7, 468. 10.3389/fchem.2019.00468 31334217 PMC6616115

[B56] Wang H. Lin C. Zhang X. Lin K. Wang X. Shen S. G. (2019a). Mussel-Inspired Polydopamine Coating: A General Strategy to Enhance Osteogenic Differentiation and Osseointegration for Diverse Implants. ACS Appl. Mater. Inter. 11, 7615–7625. 10.1021/acsami.8b21558 30689334

[B57] Wang J.-l. Li B.-c. Li Z.-j. Ren K.-f. Jin L.-j. Zhang S.-m. (2014). Electropolymerization of Dopamine for Surface Modification of Complex-Shaped Cardiovascular Stents. Biomaterials 35, 7679–7689. 10.1016/j.biomaterials.2014.05.047 24929615

[B58] Wang X. Chen Z. Yang P. Hu J. Wang Z. Li Y. (2019b). Size Control Synthesis of Melanin-like Polydopamine Nanoparticles by Tuning Radicals. Polym. Chem. 10, 4194–4200. 10.1039/c9py00517j

[B59] Wang X. Zhao H. Liu Z. Wang Y. Lin D. Chen L. (2021). Polydopamine Nanoparticles as Dual-Task Platform for Osteoarthritis Therapy: A Scavenger for Reactive Oxygen Species and Regulator for Cellular Powerhouses. Chem. Eng. J. 417, 129284. 10.1016/j.cej.2021.129284

[B60] Wang Z. Li P. Jiang Y. Jia Z. Tang P. Lu X. (2017a). Mussel-inspired Nanostructured Coatings Assembled Using Polydopamine Nanoparticles and Hydroxyapatite Nanorods for Biomedical Applications. Biosurface and Biotribology 3, 1–10. 10.1016/j.bsbt.2017.01.001

[B61] Wang Z. Wang K. Zhang Y. Jiang Y. Lu X. Fang L. (2016). Protein-Affinitive Polydopamine Nanoparticles as an Efficient Surface Modification Strategy for Versatile Porous Scaffolds Enhancing Tissue Regeneration. Part. Part. Syst. Charact. 33, 89–100. 10.1002/ppsc.201500187

[B62] Wang Z. Xie Y. Li Y. Huang Y. Parent L. R. Ditri T. (2017b). Tunable, Metal-Loaded Polydopamine Nanoparticles Analyzed by Magnetometry. Chem. Mater. 29, 8195–8201. 10.1021/acs.chemmater.7b02262

[B63] Wei Q. Zhang F. Li J. Li B. Zhao C. (2010). Oxidant-induced Dopamine Polymerization for Multifunctional Coatings. Polym. Chem. 1, 1430. 10.1039/c0py00215a

[B64] Xiang Y. Mao C. Liu X. Cui Z. Jing D. Yang X. (2019). Rapid and Superior Bacteria Killing of Carbon Quantum Dots/ZnO Decorated Injectable Folic Acid-Conjugated PDA Hydrogel through Dual-Light Triggered ROS and Membrane Permeability. Small 15, e1900322. 10.1002/smll.201900322 31021489

[B65] Xu J. Xu N. Zhou T. Xiao X. Gao B. Fu J. (2017). Polydopamine Coatings Embedded with Silver Nanoparticles on Nanostructured Titania for Long-Lasting Antibacterial Effect. Surf. Coat. Technol. 320, 608–613. 10.1016/j.surfcoat.2016.10.065

[B66] Yan J. Yang L. Lin M. F. Ma J. Lu X. Lee P. S. (2013). Polydopamine Spheres as Active Templates for Convenient Synthesis of Various Nanostructures. Small 9, 596–603. 10.1002/smll.201201064 23117928

[B67] Ye Y. Zheng L. Wu T. Ding X. Chen F. Yuan Y. (2020). Size-Dependent Modulation of Polydopamine Nanospheres on Smart Nanoprobes for Detection of Pathogenic Bacteria at Single-Cell Level and Imaging-Guided Photothermal Bactericidal Activity. ACS Appl. Mater. Inter. 12, 35626–35637. 10.1021/acsami.0c07784 32657116

[B68] Yeroslavsky G. Lavi R. Alishaev A. Rahimipour S. (2016). Sonochemically-Produced Metal-Containing Polydopamine Nanoparticles and Their Antibacterial and Antibiofilm Activity. Langmuir 32, 5201–5212. 10.1021/acs.langmuir.6b00576 27133213

[B69] Yu B. Wang D. A. Ye Q. Zhou F. Liu W. (2009). Robust Polydopamine Nano/microcapsules and Their Loading and Release Behavior. Chem. Commun., 6789–6791. 10.1039/b910679k 19885480

[B70] Zhu D. Tao W. Zhang H. Liu G. Wang T. Zhang L. (2016). Docetaxel (DTX)-loaded Polydopamine-Modified TPGS-PLA Nanoparticles as a Targeted Drug Delivery System for the Treatment of Liver Cancer. Acta Biomater. 30, 144–154. 10.1016/j.actbio.2015.11.031 26602819

